# The complete chloroplast genome sequence of *Angelica laevigata* Fisch.

**DOI:** 10.1080/23802359.2021.1958081

**Published:** 2021-07-27

**Authors:** Weichao Ren, Nannan Xing, Song Yan, Honggang Wang, Wei Ma

**Affiliations:** a Pharmacy College, Heilongjiang University of Chinese Medicine, Harbin, China; b Yichun Branch of Heilongjiang Academy of Forestry, Yichun, China

**Keywords:** *Angelica laevigata*, phylogeny, complete chloroplast genome, Umbelliferae

## Abstract

*Angelica laevigata* (Fisch 1812) is an important medicinal plant endowed with a rich chemical composition. In the present study, we present the complete chloroplast genome sequence of *A. laevigata*. The total length was 146,161 bp, comprising a large single-copy region of 93,538 bp and a small single-copy region of 17,779 bp separated by two inverted repeats of 17,422 bp each. A total of 128 genes were identified containing 87 protein-coding genes, 33 tRNA genes, and 8 rRNA genes. Phylogenetic analysis suggests that *A. laevigata* is closely associated with *Angelica laxifoliata* from the Umbelliferae family.

*Angelica laevigata* (Fisch 1812) refers to a perennial plant belonging to the Umbelliferae family. It is native to the Northeast region of China, Mongolia, the Russian Far East, and the Korean peninsula. It contains a variety of volatile compounds and a small number of coumarins, making it become an important medicinal plant (Suleimen et al. 2014). Till the present, most of the studies on this species have been mainly concentrated on describing its morphological variation and chemical composition while molecular and evolutionary researches on *A. laevigata* are lacking (Liao et al. 2013). In the current work, we provide a complete chloroplast genome sequence of *A. laevigata* which is of great necessity to comprehend its phylogenetic relationships with other Umbelliferae species.

The fresh green leaves of *A. laevigata* were collected from Yichun, Heilongjiang Province, China (N:48°22′55′′, E:129°27′95′′). The plant materials and voucher herbarium specimen were stored at the Pharmacy College of Heilongjiang University of Chinese Medicine (http://yxy.hljucm.net/, Weichao Ren, lzyrenweichao@126.com) under the voucher number YCL20200531T7. Besides, total genomic DNA including nuclear and organelle genome was extracted by employing the CTAB method (Doyle 1987; Yang et al. 2014) and was stored at Heilongjiang University of Chinese Medicine, Harbin, China. The qualified DNA was adopted to construct a 150 bp paired-end library for sequencing via Illumina NovaSeq high-throughput Sequencing platform (Benagen, Wuhan, China). The raw data was filtered using SOAPnuke (version:1.3.0) (Chen et al. 2018). Subsequently, 4.5 Gb of clean data was *de novo* assembled into circular contigs by SPAdes (version:3.13.0) (Bankevich et al. 2012). PGA (version:1) (Qu et al. 2019) was used to conduct chloroplast genome annotation as well as predict gene encoding proteins, transfer RNA (tRNA), and ribosomal RNA (rRNA). Together with gene annotation, the complete chloroplast genome sequence has been submitted to GenBank under the accession number MW696157.

Results demonstrated that the total length of *A. laevigata* chloroplast genome was 146,161 bp. Based on a typical quadripartite structure, a pair of inverted repeats (17,422 bp) was separated by a small single-copy region of 17,779 bp and a large single-copy region of 93,538 bp. The chloroplast genome of *A. laevigata* consisted of 128 genes including 87 protein-coding genes, 8 rRNA genes, and 33 tRNA genes.

To clarify the phylogenetic relationship of *A. laevigata*, the complete chloroplast genome sequences of *A. laevigata* and those from 15 related species were collected. Additionally, the chloroplast genome sequence of *Arabidopsis thaliana* was adopted as an outgroup. All sequences were aligned with the use of MAFFT (version:7.307) (Katoh and Standley 2013), followed by the construction of a phylogenetic tree obtained from a neighbor-joining (NJ) analysis with 1000 bootstraps in MEGA 7.0 (Kumar et al. 2016). The NJ phylogenetic tree revealed the close relationship of *A. laevigata* with *Angelica laxifoliata*, a member of the Umbelliferae family (Figure 1). This published chloroplast genome will be useful data for phylogenetic and evolutionary studies in Umbelliferae.

**Figure 1.**
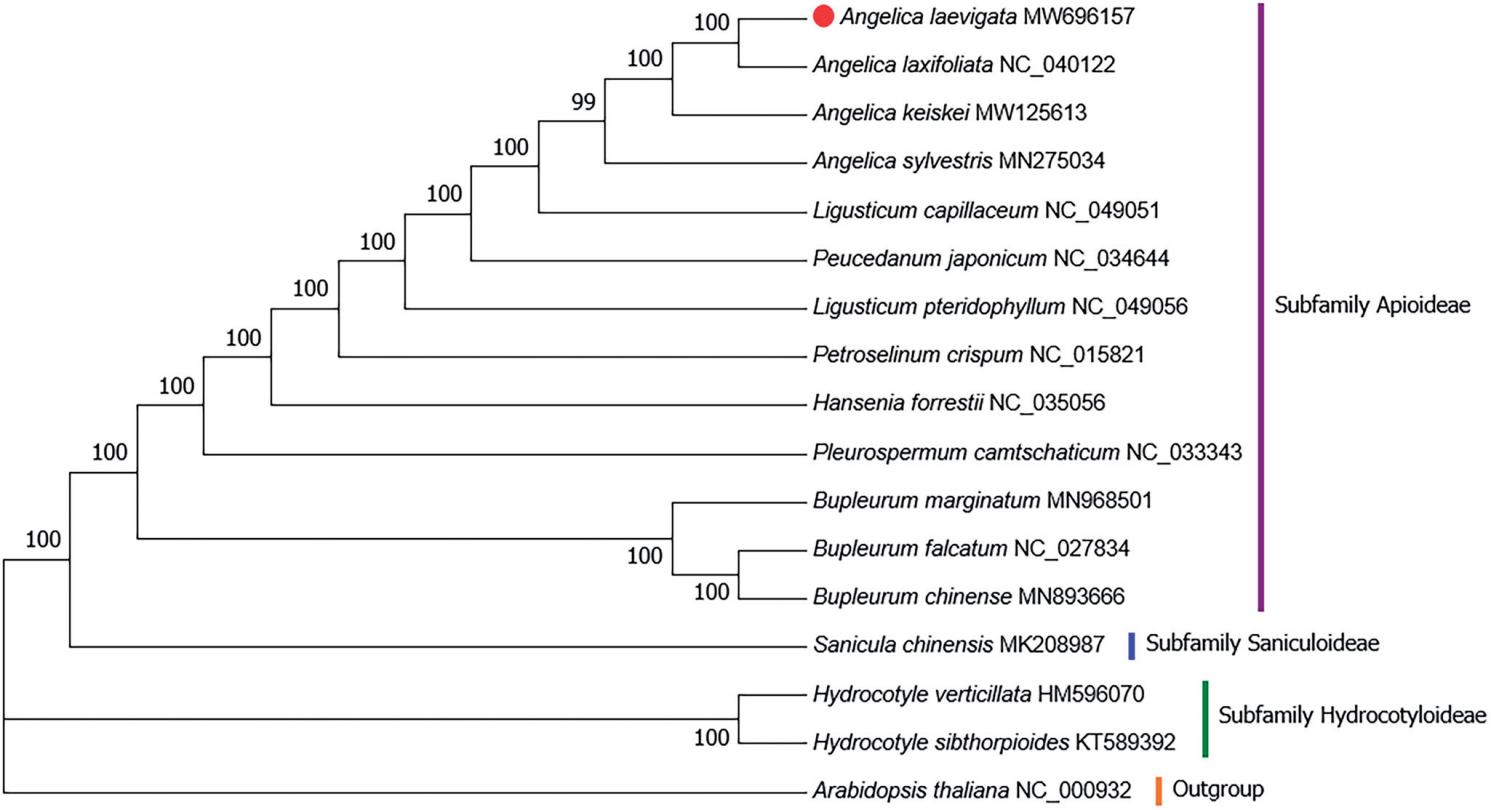
The phylogenetic tree was constructed based on whole chloroplast genome sequences of 16 Umbelliferae species and Arabidopsis thaliana as outgroup by Neighbor-Joining(NJ) method with bootstrap values from 1,000 replicates.

## Data Availability

The genome sequence data that support the findings of this study are openly available in GenBank of NCBI at (https://www.ncbi.nlm.nih.gov/) under the accession no. MW696157. The associated BioProject, SRA, and Bio-Sample numbers are PRJNA733316, SRR14723676, and SAMN19374476, respectively (https://www.ncbi.nlm.nih.gov/sra/SRR14723676).

## References

[CIT0001] Bankevich A, Nurk S, Antipov D, Gurevich AA, Dvorkin M, Kulikov AS, Lesin VM, Nikolenko SI, Pham S, Prjibelski AD, et al. 2012. SPAdes: a new genome assembly algorithm and its applications to single-cell sequencing. J Comput Biol. 19 (5):455–477. 22506599 10.1089/cmb.2012.0021 PMC3342519

[CIT0002] Chen Y, Chen Y, Shi C, Huang Z, Zhang Y, Li S, Li Y, Ye J, Yu C, Li Z, et al. 2018. SOAPnuke: a MapReduce acceleration-supported software for integrated quality control and preprocessing of high-throughput sequencing data. Gigascience. 7(1):1–6. 10.1093/gigascience/gix120 PMC5788068 29220494

[CIT0003] Doyle J. 1987. A rapid DNA isolation procedure for small quantities of fresh leaf tissue. Phytochem Bull. 19:11–15.

[CIT0004] Katoh K, Standley D. 2013. MAFFT multiple sequence alignment software version 7: improvements in performance and usability. Mol Biol Evol. 30(4):772–780. 23329690 10.1093/molbev/mst010 PMC3603318

[CIT0005] Kumar S, Stecher G, Tamura K. 2016. MEGA7: Molecular Evolutionary Genetics Analysis version 7.0 for bigger datasets. Mol Biol Evol. 33(7):1870–1874. 27004904 10.1093/molbev/msw054 PMC8210823

[CIT0006] Liao C, Downie SR, Li Q, Yu Y, He X, Zhou B. 2013. New insights into the phylogeny of *Angelica* and its allies (Apiaceae) with emphasis on east Asian species, inferred from nrDNA, cpDNA, and morphological evidence. Syst Bot. 38(1):266–281.

[CIT0007] Qu XJ, Moore MJ, Li DZ, Yi TS. 2019. PGA: a software package for rapid, accurate, and flexible batch annotation of plastomes. Plant Meth. 15(1):1–12. 10.1186/s13007-019-0435-7 PMC6528300 31139240

[CIT0008] Suleimen EM, Iskakova ZB, Salokhin AV, Gorovoi PG, Wang M, Khan I, Ross SA. 2014. Compositions of essential oils from *Angelica czernaevia* and a. *ursina* growing in the Russian far east. Chem Nat Compd. 50(4):750–752.

[CIT0009] Yang JB, Li DZ, Li HT. 2014. Highly effective sequencing whole chloroplast genomes of angiosperms by nine novel universal primer pairs. Mol Ecol Resour. 14(5):1024–1031. 24620934 10.1111/1755-0998.12251

